# Dangers of Using Bee Propolis and Foeniculum Vulgare Before Browplasty

**DOI:** 10.7759/cureus.19909

**Published:** 2021-11-25

**Authors:** Afaf Shaabi, Eman Badawi

**Affiliations:** 1 Neurological Surgery, King Fahad General Hospital, Jeddah, SAU; 2 Oculoplastic, Jeddah Eye Hospital, Jeddah, SAU

**Keywords:** ­wound healing, browplasty, coagulopathy, propolis, foeniculum vulgare

## Abstract

Several medicinal herbs have been associated with coagulopathy. They can be readily purchased at a local herb store without prescription or expert opinion based on scientific evidence. This is probably why patients do not disclose using these herbs to health care providers even on direct questioning. Here, we focus on two commonly used herbs in our community, bee propolis, and *Foeniculum vulgare*, also known as fennel seeds, as our patient ingested them before a scheduled browplasty. The significance of this anecdote is the concomitant ingestion of fennel seeds and bee propolis. Arguably, their consumption resulted in excessive intraoperative and postoperative bleeding in a previously healthy subject. This complication is undesirable in any aesthetic surgery, as it affects wound healing and the final cosmetic outcome. We hope that such bioactive herbs are warned against and that their risks are known to patients and physicians alike.

## Introduction

The availability and usage of medicinal herbs have grown tremendously among the Saudi population in recent years [1]. They are allegedly safe [2], and the Saudi population eagerly advocates for their integration into primary health care services [3]. However, herb-induced perioperative coagulopathy, in particular, is a serious side effect that invariably affects the outcome in any type of surgery, especially aesthetic [4].

In vivo, clinical trials have shown that fennel seeds (*Foeniculum vulgare*), which are mostly composed of volatile oil (anethole), exhibited potent antiplatelet activity and clot destabilization [5]. Likewise, propolis has shown a preventive effect on in vitro platelet aggregation [6]. Both products are licensed in Saudi markets as uncontrolled over-the-counter dietary supplements [7]. More information about the manufacturing details can be found at the registered drugs and herbal products list released by the Saudi Food and Drug Authority. Interestingly, a routine preoperative coagulation profile would not detect the effect of these supplements. Because the prothrombin time and activated partial thromboplastin time, which reflect the extrinsic and intrinsic pathways of coagulation, respectively, would probably be within the normal range [8].

## Case presentation

A healthy 45-year-old woman presented to our oculoplastic clinic complaining of droopy eyebrows. After complete history and examination, she was found to be a good candidate for bilateral direct browplasty.

An initial superficial incision was made inside the upper border of the right brow with a #15 blade. Next, unexpected capillary bleeding at the surgical site evenly from the subcutaneous fat was found difficult to control. No major blood vessel was transected, as the incision was superficial. Several minutes later, after applying pressure, we achieved homeostasis using monopolar electrocautery with one layer interrupted 4.0 nonabsorbable suture. Thereon, the lead surgeon opted for postponing the contralateral browplasty to a later date.

The decision to postpone the contralateral browplasty was most likely because our patient had been ingesting fennel seeds and propolis for months. New information became known on the antiplatelet effect of both herbs in a quick Google scholar search. The team suggested two weeks of abstinence to eliminate any potential coagulopathy caused by these two herbs.

On the fourth postoperative day, as suture removal resulted in prolonged bleeding duration, some sutures were left in situ for a longer time duration. Contralateral browplasty was performed after 14 days, and it went uneventfully. Wound examination in the weekly follow-up visit did not show any signs of poor healing or surgical site infection. The remaining sutures were removed without complications with an acceptable final cosmetic outcome.

## Discussion

We describe a patient who developed more than the normal estimated intraoperative bleeding from a browplasty incision. We initially considered two reasons to explain this unforeseen incident. First, we thought of drug-induced coagulopathy, but her medical history did not support this claim. Second, because coagulation profiles are not tested routinely in our hospital as part of the preoperative assessment, we could not exclude intrinsic coagulopathy at first. However, complete medical history and examination did not reveal bleeding tendencies to prompt a laboratory test.

On direct questioning, the patient disclosed daily drinking of steeped fennel seeds and purified propolis in oral capsules (unknown concentration). She purchased them from a local herb store without proper counseling from a certified health care provider. In Saudi markets, bee propolis is sold as an oral liquid and capsule formulas purified with other active substances. *Foeniculum vulgare* is classified as a condiment and is available as whole seeds and in powdered form.

These supplements are advertised as natural blood pressure-lowering agents. Our patient included them in her daily diet after receiving a probable diagnosis of hypertension from her primary physician. In recent in vivo clinical trials, fennel seeds promoted vasodilation [5]. Noticeably, the patient’s blood pressure readings were within normal limits at preoperative assessment and clinic visits. She stopped ingesting these herbs on advice from her dentist, who had encountered a similar complication during a tooth extraction procedure months ago. The patient concealed this information on the assumption that 48 hours is enough time to eliminate the risk of intraoperative bleeding. The critical fact may be the co-consumption of herbs, both of which have proven antiplatelet activity. This fact leads to the core issue of dietary supplements having similar or perhaps synergistic effects, such as the antiplatelet effect. Most likely, these supplements are dispensed without warning against their concomitant use. We have not come across research that studied the possible interaction between bee propolis and *Foeniculum vulgare* in particular. However, their biochemical properties are highly suggestive of similar or even synergistic effects.

Two weeks later, contralateral browplasty was performed, and we did not encounter prolonged bleeding. Since abstaining from fennel seeds and bee propolis ingestion had corrected her coagulopathy, it is unlikely that she had an intrinsic coagulopathy. Nevertheless, she was advised to follow up with her primary physician to diagnose and manage hypertension.

## Conclusions

This article is written to raise awareness among surgeons about the detrimental and hemorrhagic potential of fennel seeds and bee propolis. The goal is to improve the rate of detecting patients consuming these herbs in the preoperative assessment before any major or minor surgical procedure. We also highlight the importance of restricting the usage of these supplements under medicinal laws. Since the full extent of their effects on our physiology is still unknown, large-scale controlled clinical trials are needed to investigate the biochemical properties of supplements such as the ones described here and especially their consequences in the critical perioperative period.

## References

[1] Al Akeel MM, Al Ghamdi WM, Al Habib S, Koshm M, Al Otaibi F (2018). Herbal medicines: Saudi population knowledge, attitude, and practice at a glance. J Fam Med Prim Care.

[2] Bogusz MJ, al Tufail M, Hassan H (2002). How natural are 'natural herbal remedies'?: a Saudi perspective. Adv Drug React Toxicol Rev.

[3] Allam S, Moharam M, Alarfaj G (2014). Assessing patients' preference for integrating herbal medicine within primary care services in Saudi Arabia. J Evid Based Compl Altern Med.

[4] Wong WW, Gabriel A, Maxwell GP, Gupta SC (2012). Bleeding risks of herbal, homeopathic, and dietary supplements: a hidden nightmare for plastic surgeons?. Aesthet Surg J.

[5] Tognolini M, Ballabeni V, Bertoni S, Bruni R, Impicciatore M, Barocelli E (2007). Protective effect of Foeniculum vulgare essential oil and anethole in an experimental model of thrombosis. Pharmacol Res.

[6] Zhang YX, Yang TT, Xia L, Zhang WF, Wang JF, Wu YP (2017). Inhibitory effect of propolis on platelet aggregation in vitro. J Healthc Eng.

[7] (2020). Saudi Food and Drug Authority: Registered drugs and herbal products list. https://www.sfda.gov.sa/ar/drug/search/Pages/default.aspx?PageIndex=1&sm=herbal.

[8] Sugita C, Yamashita A, Tsutsumi S (2021). Brazilian propolis (AF-08) inhibits collagen-induced platelet aggregation without affecting blood coagulation. J Nat Med.

